# Effects of Defatting Pretreatment on Polysaccharide Extraction from Rambutan Seeds Using Subcritical Water: Optimization Using the Desirability Approach

**DOI:** 10.3390/foods13131967

**Published:** 2024-06-21

**Authors:** Kamonthip Nilmat, Panusorn Hunsub, Somkiat Ngamprasertsith, Winatta Sakdasri, Aphichart Karnchanatat, Ruengwit Sawangkeaw

**Affiliations:** 1 Program in Biotechnology, Faculty of Science, Chulalongkorn University, 254 Phayathai Road, Pathumwan, Bangkok 10330, Thailand; 2 Fuels Research Center, Department of Chemical Technology, Faculty of Science, Chulalongkorn University, 254 Phayathai Road, Pathumwan, Bangkok 10330, Thailand; 3 Program in Food Process Engineering, School of Food Industry, King Mongkut’s Institute of Technology Ladkrabang, 1 Chalong Krung 1 Alley, Latkrabang, Bangkok 10520, Thailand; 4 Center of Excellence in Bioconversion and Bioseparation for Platform Chemical Production, Institute of Biotechnology and Genetic Engineering, Chulalongkorn University, 254 Phayathai Road, Pathumwan, Bangkok 10330, Thailand

**Keywords:** polysaccharides, monosaccharides, total sugar content, rambutan seed, subcritical water extraction, response surface methodology, supercritical fluid extraction

## Abstract

Rambutan seeds are by-products generated from fruit-processing factories; the leftover seeds are buried in landfills, generating methane emissions. This work aimed to extract polysaccharides (POLS) from rambutan seeds by using subcritical water extraction (SWE). The effects of defatting pretreatment and operating parameters in SWE were investigated using a Box–Behnken design. The results show that defatting pretreatment significantly enriched the POLS yield, while it had no significant effect on the total sugar content. Using the desirability approach, the suitable feedstock for SWE was defatted rambutan seeds. The maximum desirability of 0.86 was found at a temperature range of 145–150 °C, an extraction time of 15 min, and a liquid–solid ratio of 10:1. The POLS yield and total sugar content were in the range of 52.33–55.63 g/100 g feedstock and 83.37–87.45 g/100 g POLS, respectively. The extracted POLS had an equivalent molecular weight of 413.70 kDa that could be used as an extender in plant-based products. In conclusion, the defatting pretreatment of rambutan seeds not only improved the POLS yield obtained via SWE but also generated additional lipids that could be utilized as an unconventional source of specialty fat.

## 1. Introduction

The rambutan fruit (*Nephelium lappaceum*) is a tropical fruit native to Southeast Asia. In Thailand, the rambutan output is approximately 218.6 thousand tons per year, and 11.85 thousand tons of rambutan were exported at a cost of around USD 7.2 million in 2021–2022 [1]. Rambutan is industrially processed into canned fruit, juice, jam, and spread, which generates seed waste that is approximately 10% of the fresh fruit’s weight or 26,592 tons annually [2]. The nutritional compositions of rambutan seeds have been reported in many literatures [3,4,5,6,7,8]. Rambutan seeds contain high amounts of carbohydrates (28.7–62.4 g/100 g) and lipids (28.20–41.30 g/100 g). Mexican rambutan seeds have the lowest protein and ash contents of 7.80 and 1.22 g/100 g, respectively [3]. Malaysian rambutan seeds comprise the highest protein content in the range of 12.40–13.70 g/100 g [5,6]. It was reported that Thai rambutan seeds have the highest lipid content in the range of 37.35–41.30 g/100 g [7,8].

In previous work, the green extraction technologies employed for extracting oil from rambutan seeds were a mechanical screw press and supercritical carbon dioxide–ethanol extraction, called defatting pretreatment hereafter. The advantages of mechanical extraction include the ease of use, a quick extraction cycle, versatility in oilseed application, and a low operating cost [9]. However, mechanical extraction encounters the problems of low extraction yield and low purity of the extracted oil.

For supercritical fluid extraction, environmental benefits such as operation without hazardous organic solvents and usage of non-flammable CO_2_ as a solvent are the major strong points, while the high instrument cost and high-pressure operation are the drawbacks of this technique [10]. It has been reported that the extracted lipid from rambutan seeds is a promising unconventional source of specialty fat for the cosmetic and personal care industries because it contains oleic and arachidic acids as the major fatty acids [11]. In our previous work, rambutan seed fat was used for the formulation of a biocomposite film. It was found that the addition of rambutan seed fat reduced water absorption and improved water vapor permeability [12].

After the defatting pretreatment was complete, polysaccharides (POLS) were sequentially extracted from the defatted rambutan seeds using the subcritical water extraction (SWE) technique. Among various polysaccharide extraction methods such as hot water extraction, alkaline water extraction, ultrasonic-assisted extraction, microwave-assisted extraction, enzyme-assisted extraction, and ultra-high-pressure extraction, SWE was selected because of the process’s simplicity and the availability of instrument in our research group [13,14,15]. Furthermore, SWE has been promoted as an efficient technology for extracting polysaccharides [16]. Finally, as noted in recent review articles [17,18], there is no report on the extraction of POLS from rambutan seeds using SWE.

POLS are complex carbohydrates that contain monosaccharides in the forms of homopolysaccharide and heteropolysaccharide, such as starch, cellulose, pectin, and gums. The monosaccharide composition of plant seeds includes galactose, xylose, glucose, arabinofuranose, and galactopyranose [19]. Currently, POLS are used in the food industry as food additives with antioxidant properties, emulsifiers, and gel-forming substances. Research on the nutrients and medicinal effects of POLS obtained from seeds has found them to have antioxidant, anti-cancer, immune-modulating, anti-aging, and blood-sugar-lowering properties [20,21].

In this work, we hypothesized that defatting pretreatment would improve the effectiveness of SWE in terms of the POLS yield and total sugar content. This hypothesis was tested using response surface methodology and optimized using the desirability approach. The numerical parameters were the temperature, extraction time, and liquid–solid ratio, while the categorical parameters were the feedstock type, dried rambutan seed (RS), and defatted rambutan seed (DRS). The responses were the POLS yield and total sugar content. The process optimization was conducted using the desirability function (*d_i_*) [22]. Obtaining the maximum POLS yield and total sugar content was set as the goal of optimization. The weight and importance of both responses were set to be equal. The values of *d_i_* varied in the interval of 0 ≤ *d_i_* ≤ 1, while the overall desirability (*D*) was calculated based on the geometric mean [23]. The temperature and extraction time were minimized at the initial stage of optimization. Finally, the extracted POLS obtained under the optimal conditions were examined with regard to the equivalent molecular weight and monosaccharide profile.

## 2. Materials and Methods

### 2.1. Raw Material and Reagents

Rambutan seed waste was donated by Pissanumhon Food Products Company Limited, Chumphon province, Thailand, from April to July 2023. Carbon dioxide (99.8%) was supplied by Linde Co., Ltd. (Samut Prakan, Thailand). Hydrated ethanol (95.5%) and absolute ethanol (99.5%) were purchased from the Liquor Distillery Organization, Thailand. Methanol of analytical grade (99.9%) was purchased from RCI Labscan. Phenol GR for analysis and sulfuric acid were supplied by Merck (Darmstadt, Germany). Galactose (Gal), arabinose (Ara), rhamnose (Rha), glucose (Glc), xylose (Xyl), and mannose (Man) were supplied by Sigma (St. Louis, MO, USA).

### 2.2. Apparatus

A single-screw-press machine (Model YZYX70-ZWY) was purchased from Mianyang Guang Xin Machinery Factory Co., Ltd., Guangzhou, China. The supercritical CO_2_ extractor was supplied by Agricultural Engineering Research Institute, Department of Agriculture, Ministry of Agriculture and Cooperatives (Khon Kaen, Thailand). Further details of the screw-press machine and supercritical CO_2_ extractor have been described in previous works [24,25]. A household blender (Model MX-AC400, Panasonic Co., Ltd., Bangkok, Thailand) and a microplate reader (Thermo Multiskan FC, Thermo Fisher Scientific Inc., Waltham, MA, USA) were purchased from a local supplier. The subcritical water extractor was supplied by Parr company (Moline, IL, USA), Series 4625. The high-performance liquid chromatograph (HPLC) was supplied by Shimadzu, Co., Ltd. (Kyoto, Japan).

### 2.3. Feedstock Preparation and Characterization

The fresh rambutan seeds were cleaned and washed with running tap water. Then, seeds were dried in a hot air oven at 60 °C for 8 h. The dried rambutan seeds were ground with a blender, called rambutan seed powder (RS) hereafter. A total of 50 kg of whole dried rambutan seeds was pre-extracted without any size reduction step using a screw-press machine at a constant rotating speed of 25 rpm. The extraction temperature was controlled to be 80 °C during operation by manually alternating the feed rate between 3 kg/h and 5 kg/h and the screw/barrel clearance. Then, the screw-pressed cakes were defatted via supercritical CO_2_–ethanol extraction. The maximum CO_2_ flow rate and working pressure were 200 L/h and 35.0 MPa, respectively. A total of 2 kg of screw-pressed cakes was soaked in 2 kg of 95.5% ethanol under 30 MPa and a temperature of 50 ± 5 °C for 90 min. The ethanol to CO_2_ mass ratio was 1:8 in all operations. The defatted rambutan seed (DRS) was obtained after being dried at 27 °C for 24 h. Both RS and DRS were sieved to obtain a particle size range of 1–3 mm before characterization and extraction. Proximate analysis of RS and DRS was performed based on the Association of Official Analytical Chemist (AOAC international) standard methods [26].

### 2.4. Subcritical Water Extraction of RS and DRS

A total of 10 g of the sample (RS or DRS) was extracted in a high-pressure batch reactor (working volume of 500 mL). The reactor was purged and pressurized with nitrogen at a pressure of 2 MPa prior to increasing the temperature. The extraction conditions followed the Box–Behnken design, which consisted of 28 experimental points, as shown in Table 1. After the extraction was complete, the reactor was cooled in an ice-water bath at 4 °C for 10 min. Afterwards, the extract was separated from the solid residue using a paper filter. The extracted polysaccharides were purified by means of precipitation with cold 95% ethanol at an extract-to-ethanol ratio of 1:4 (*v*/*v*). The precipitate was collected after the mixture was left at 4 °C for 12 h and centrifuged at 5000 rpm and 4 °C for 20 min [27]. The solid sample was dried in a hot air oven at 40 °C for 12 h. The crude polysaccharide (POLS) yields were calculated according to Equation (1):

Y_1_ (%) = (W_p_/W_s_) × 100 (1)

where Y_1_ is the polysaccharide (POLS) yield (%); W_p_ is the weight of the dried POLS; and W_s_ is the weight of the sample.

### 2.5. Analysis of Total Sugar Content in POLS

The total sugar content was analyzed using the phenol–sulfate assay [27]. A total of 0.1 mg of POLS powder was mixed with 100 µL of a phenol aqueous solution (5% *w*/*v*) and 500 µL of concentrated sulfuric acid. D-glucose was used as the reference standard. The mixture was incubated in the dark at room temperature for 30 min. Afterwards, the absorbance was measured using a microplate reader at 490 nm.

### 2.6. Size Distribution of POLS

The molecular size distribution of the POLS obtained under the optimal conditions was determined using high-performance size-exclusion chromatography (HPOLSEC) [28]. Solutions of POLS at 5 mg/mL were prepared in DDI water and filtered through a 0.45 µm nylon syringe filter. The HPOLSEC system consisted of an LC-20AD pump, an RID-10A detector, a Ultrahydrogel linear column (7.8 × 300 mm, Waters, Milford, MA, USA) with a guard column (6.0 × 40 mm), and a computer with a data analysis software program (CLASS-VP, version 5.0). The injection volume was 20 µL. The isocratic elution using 0.1 M sodium nitrate (containing 0.02% NaN_3_ as a preservative) as the mobile phase was performed at a temperature of 60 °C and a flow rate of 0.8 mL/min. Pullulan polysaccharides were used as the reference standards with average molecular weights of 5.9, 11.8, 22.8, and 788.0 kDa.

### 2.7. Monosaccharide Profile of POLS

The monosaccharide profile of POLS was analyzed using high-performance liquid chromatography (HPLC) [28]. A total of 10 mg of the POLS samples was hydrolyzed with 1 mL of 0.5 M H_2_SO_4_ at 100 °C for 3 h. Subsequently, the solution was cooled to room temperature and its pH was adjusted to 7.0 using 2 M NaOH. After that, the samples were diluted in 5 mL of DDI water and filtered through a 0.45 µm nylon syringe filter. The HPLC system was similar to the system described in Section 2.5. The analytical column was a VertiSep OA 8 μm column (7.8 × 300 mm) and the mobile phase was 3% acetonitrile in 0.75 mM H_2_SO_4_ at a flow rate of 0.5 mL/min. [28]. Glucose, galactose, arabinose, rhamnose, and xylose were used as the reference standards.

### 2.8. Experimental Design and Statistical Analysis

The Box–Behnken design was used to examine the effects of temperature, time, liquid–solid ratio, and feedstock type on the POLS extraction yield and total sugar content. The experiments were conducted in duplicate for the RS and DRS, as shown in Table 1. Statistical analysis was performed using Design-Expert 13.0 (Stat-Ease Inc., Minneapolis, MN, USA) for Windows.

## 3. Results and Discussion

### 3.1. Proximate Analysis

Table 2 depicts the nutritional composition of the RS and DRS obtained from proximate analysis as mentioned in Section 2.3. The major fatty acids of rambutan seed lipid were oleic (C18:1) and arachidic (C20:0) acids (see Table S1), which agreed well with the literature. The carbohydrate content in the DRS was higher than in the RS because the defatted process reduced the fat content in the RS. The moisture content of the DRS was also significantly reduced because mechanical extraction via a screw-press machine enhanced the surface area of the RS and facilitated water evaporation. On the other hand, the contents of protein, crude fiber, and ash in both the RS and DRS were not significantly different. Thus, the removal of lipids and moisture did not impact those nutrients.

### 3.2. Effect of Operating Parameters on POLS Yield

**Figure 1:**
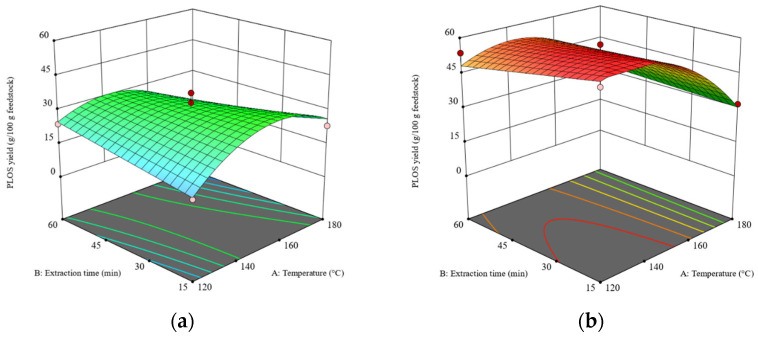
The 3D surface plots at a liquid–solid ratio of 20:1 (*w*/*w*): (**a**) the effects of temperature and time on the POLS yield obtained from the RS and (**b**) the effects of temperature and time on the POLS yield obtained from the DRS. The z-axis of both plots was adjusted to similar minimum and maximum values.

The results of the analysis of variance (ANOVA) of the POLS extraction yield are shown in Table S2. The *p*-value of the model is 0.0010 and the *p*-value of the lack-of-fit test is 0.1606, indicating that the model is significant (*p* ≤ 0.05). These results indicate that the model can effectively predict the POLS yield extracted from the rambutan seeds. The results show that the main factors affecting the POLS yield are temperature (X_1_) and feedstock types (X_4_). After eliminating insignificant factors via the multimodel selection methods, setting the criterion with the *p*-value set at an alpha of 0.1 [23], and including the hierarchical terms, the reduced cubic model was obtained, as shown in Equation (2). The 3D response surface plots of the POLS yields obtained from the RS and DRS are shown in Figure 1a,b, respectively.

Y_1_ = 42.99 − 5.83 X_1_ − 1.79 X_2_ + 1.18 X_3_ − 11.82 X_4_ − 1.50 X_1_X_2_ + 4.99 X_1_X_3_ − 5.11 X_1_X_4_ + 6.10 X_2_X_3_ − 0.34 X_2_X_4_ − 1.24 X_3_X_4_ − 12.04 X_1_^2^− 4.41 X_3_^2^ + 4.83 X_1_X_2_X_3_ − 7.64 X_3_^2^ X_4_ (2)

Here, Y_1_ is the POLS yield (g POLS/100 g feedstock). X_1_, X_2_, X_3_, and X_4_ are the temperature, extraction time, liquid–solid ratio, and feedstock type in terms of the coded units (see Table 1), respectively.

The POLS yields obtained from the DRS were higher than those obtained from the RS because the DRS had a higher carbohydrate content. Feedstock type showed the highest sum of squares compared to the other factors in the ANOVA (see Table S2), which indicates the significant impact of the defatting pretreatment on POLS yield. The removal of the lipid barrier enhanced the efficiency of SWE, as demonstrated in the extractions of rice bran and soybean meal in previous research [29]. This work employed mechanical extraction using a screw-press machine following by supercritical CO_2_–ethanol extraction as a sequential defatting method (see Section 2.2). The CO_2_–ethanol mixture was selected because the final products, e.g., lipids, polysaccharides, and proteins were expected to be used in the food industry. In fact, the most suitable solvent is pure CO_2_. Unfortunately, using pure CO_2_ as supercritical solvent required the high pressure, >30 MPa to completely extract rambutan seed in a suitable extraction time [25]. Addition of ethanol was attempted to reduce the optimal pressure. In addition to the removal of the extracted lipid, the destruction of the RS structure by high pressure could be expected.

It has been reported that the optimal temperature for SWE of POLS is in the range of 130–240 °C [18]. Because the dielectric constant of water reduces with an increasing temperature [30], the extraction efficiency of SWE is enhanced as well. As revealed in Figure 1, the maximum POLS yields obtained from the RS and DRS were 32–34 g POLS/100 g RS and 48–52 g POLS/100 g DRS, respectively. Extraction time had no significant effect on the POLS yields. The maximum POLS yield from the RS was obtained at a temperature of 150 °C, while the maximum POLS yield from the DRS was obtained at a temperature range of 120–150 °C. Hence, it is concluded that the defatting pretreatment significantly improves the extraction of POLS from rambutan seeds.

When comparing the carbohydrate contents in the RS (42.96 g/100 g RS) and DRS (61.63 g/100 g DRS) with the POLS yields, 94% and 93% of carbohydrates in the RS and DRS, respectively, were extracted via SWE. However, when the temperature was over 150 °C for both the RS and DRS, the POLS yields dropped because the POLS were hydrolyzed to small molecules and could not be precipitated by cold ethanol.

### 3.3. Effect of Operating Parameters on Total Sugar Content in POLS-RS and POLS-DRS

**Figure 2:**
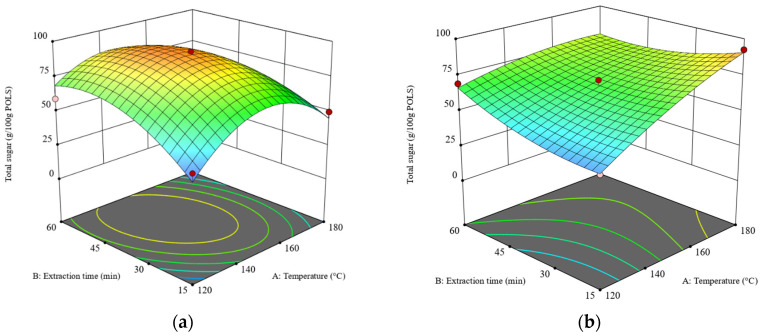
The 3D surface plots at a liquid–solid ratio of 20:1 (*w*/*w*): (**a**) the effects of temperature and time on total sugar obtained from the RS and (**b**) the effects of temperature and time on total sugar obtained from the DRS. The z-axis of both plots was adjusted to similar minimum and maximum values.

The ANOVA results of the total sugar content are presented in Table S3. The regression model is significant because it has a *p*-value less than 0.0001 and the *p*-value of the-lack-of-fit test is 0.2838. Unlike the POLS yield, all main factors and their interactions significantly affected the total sugar content. After eliminating an insignificant factor (X_2_^2^), a reduced regression model was obtained, as shown in Equation (3). Figure 2a,b depict the 3D response surface plots of the total sugar content obtained from the RS and DRS, respectively.

Y_2_ = 79.35 + 5.21X_1_ + 5.00X_2_ + 3.58X_3_ − 11.45X_4_ − 8.76X_1_X_2_ + 6.76X_1_X_3_ + 9.78X_1_X_4_ − 5.41X_2_X_3_ − 6.45X_3_X_4_ − 15.72X_1_^2^ + 7.55X_3_^2^ + 11.65X_1_^2^ X_4_ + 10.63X_2_^2^X_4_ + 7.07 X_3_^2^X_4_ (3)

Here, X_1_, X_2_, X_3_, and X_4_ are the temperature, extraction time, liquid–solid ratio, and feedstock type in terms of the coded units (See Table 1), respectively. Y_2_ is the total sugar content (g/100 g POLS).

As shown in Figure 2, the 3D surface plots of total sugar obtained from the RS and DRS are concave (dome-like) and semi-flat shapes, respectively. Hence, the stationary point of Figure 2a is the maximum value, while the stationary point of Figure 2b is at the edge of the experimental condition [31]. For SWE of the RS, the maximum total sugar content was obtained at a temperature range of 140–160 °C and an extraction time of 30–60 min at a liquid–solid ratio of 20:1 (*w*/*w*). For SWE of the DRS at a constant extraction time of 15 min, increasing the temperature from 120 °C to 180 °C enhanced the total sugar content by over three folds. However, at an extraction time of 60 min, the effects of temperature on total sugar content declined because the system reached the extraction equilibrium [30]. These results indicate that the defatting pretreatment differentiates the SWE of rambutan seeds.

### 3.4. Process Optimization Using the Desirability Approach

**Figure 3:**
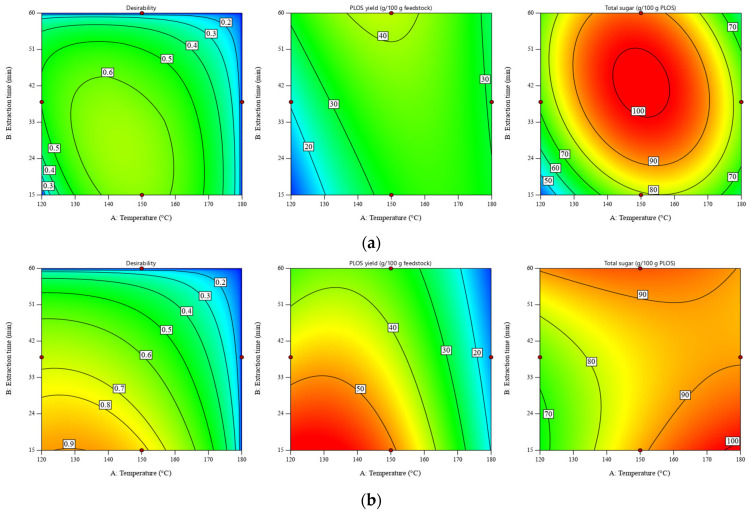
Desirability of POLS yield and total sugar content as a function of temperature and extraction time for SWE of (**a**) RS at a liquid–solid ratio of 30:1 *w*/*w* and (**b**) DRS at a liquid–solid ratio of 10:1 *w*/*w*. Maximizing both the POLS yield and total sugar content is the optimization goal. Minimizing the temperature and extraction time are the constraints.

According to our previous work [11], the maximum POLS yield was observed at a temperature of 120 °C and an extraction time of 60 min. This work extended the investigated parameters and optimized the POLS extraction by using the desirability approach. Despite the maximum POLS yield of the DRS at a temperature of 120 °C and an extraction time of 15 min (see Figure 1b), the total sugar content of POLS obtained under this condition was the lowest value, at ~20 g/100 POLS. Hence, the process optimization was conducted by using the desirability function where obtaining the maximum POLS yield and total sugar content was set as the goal. Temperature and extraction time were minimized, and the liquid–solid ratio was adjusted within the experimental range. The graphical representation of optimization using the desirability approach is shown in Figure 3.

According to Figure 3, the maximum values of desirability for SWE of the RS and DRS estimated at liquid–solid ratios of 30:1 and 10:1 were 0.66 and 0.85, respectively. It should be noted that optimization was conducted at different liquid–solid ratios because the carbohydrate contents in the RS and DRS were significantly different. More details of the numerical solutions are presented in Table S4. For the RS, the highest desirability of 0.66 was found in the temperature range of 140–145 °C and the extraction time range of 23–32 min. For the DRS, the highest POLS yield (57.69–58.86 g/100 g feedstock) and total sugar content (67.76–79.07 g/100 g POLS) were observed within the temperature range of 120–135 °C and the extraction time range of 15–16 min.

**Figure 4:**
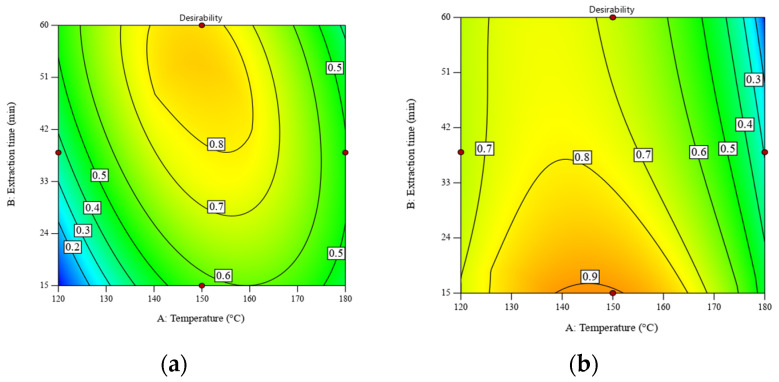
Desirability as a function of temperature and extraction time for SWE of (**a**) RS at a liquid–solid ratio of 30:1 *w*/*w* and (**b**) DRS at a liquid–solid ratio of 10:1 *w*/*w*. Maximizing both the POLS yield and total sugar content is the optimization goal. All operating parameters have no constraint.

When the constraints of temperature and extraction time were removed, the values of desirability were improved by 0.79 and 0.86, respectively. Figure 4 displays the contour plots of desirability as a function of temperature and extraction time. The desirability of the RS significantly improved from 0.66 to 0.79 when increasing the extraction time from 30 min to 60 min, while that of DRS was still stable at ~0.9. Table S5 shows the numerical solutions shown in Figure 4.

After the optimal conditions were identified, the regression model was verified by means of quintuplicate experiments within the temperature range of 145–150 °C, an extraction time of 15 min, and a liquid–solid ratio of 10:1 using the DRS as the feedstock. It was found that the POLS yield and total sugar content were within the predicted values of 52.33–55.63 g/100 g feedstock and 83.37–87.45 g/100 g POLS, respectively. The samples obtained under the optimal conditions, including (a) a temperature of 150 °C and an extraction time of 60 min for the RS (called POLS-RS hereafter) and (b) a temperature of 140 °C and an extraction time of 15 min for the DRS (called POLS-DRS hereafter), were characterized for their molecular weight distribution and monosaccharide profile, as described in Section 3.3.

### 3.5. Molecular Weight Distribution and Monosaccharide Profile of the Extracted POLS

**Figure 5:**
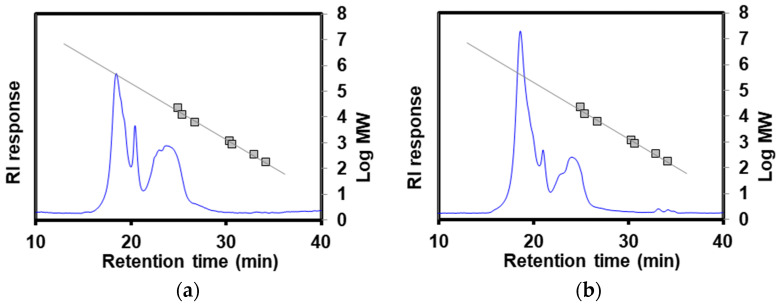
HPOLSEC chromatograms of (**a**) POLS-RS and (**b**) POLS-DRS. Linear trend lines are reference standards (pullulans).

Figure 5 depicts the chromatograms of HPOLSEC. Table 3 presents the retention time and equivalent molecular weight (MW_eq_). The values of the MW_eq_ of POLS-RS and POLS-DRS were 434.84 kDa and 413.70 kDa, respectively. These MW_eq_ values aligned with the POLS value of 420 kDa extracted from longan (in the same *Sapindaceae* family as rambutan) seeds [32]. In contrast, the MW_eq_ values of POLS-RS and POLS-DRS were significantly higher than that of POLS extracted from *Camellia oleifera* Abel (14.95–87.76 kDa) using hot water extraction at 85 °C and 130 min [33]. These results imply that the defatting pretreatment did not impact the molecular weight of the extracted POLS. These high-molecular-weight POLS could be used as a thickener for making cream or as an extender in plant-based meat analogue products [34].

As shown in Table 4, POLS-RS, which has a total sugar content of ~90 g/100 POLS, consists of 91.91% (*w*/*w*) glucose, 6.46% (*w*/*w*) galactose, 1.27% (*w*/*w*) arabinose, and 0.36% rhamnose (*w*/*w*). POLS-DRS, which has a total sugar content of ~80 g/100 POLS, is composed of 92.21% glucose (*w*/*w*), 6.68% galactose (*w*/*w*), 0.8% arabinose (*w*/*w*), and 0.2% rhamnose (*w*/*w*). It was previously reported that POLS extracted from *Litchi chinensis* Sonn. (in the same *Sapindaceae* family as rambutan) via ultrasound-assisted extraction comprised 57.3% galactose, 29.7% glucose, 6.5% mannose, 3.2% fructose, and 3.3% arabinose [35]. Further study on POLS extracted from longan seeds showed a composition of 33.7% galactose, 17.6% glucose, and 32.8% arabinose [32]. Hence, the POLS extracted from rambutan seeds via SWE show a unique property because their composition contains over 90% glucose as a major monosaccharide.

From the perspective point of view, the extent of this work would aim to extract the fresh rambutan seeds at the processing factory where the waste is generated to eliminate the logistic costs. Because of the high moisture content up to 40%wt. in fresh rambutan seeds [25], the drying process requires huge amounts of energy. However, it could be assumed that the properties of extracted lipids obtained from fresh rambutan are different from those extracted from dried rambutan seed. Furthermore, this process could estimate the profitability and environmental impact by using a techno-economic analysis and life-cycle assessment in future study.

## 4. Conclusions

Defatting pretreatment via mechanical and supercritical water extraction improved the POLS yield and total sugar content of rambutan seeds in two ways. First, the defatting pretreatment increased the carbohydrate content in the substrate by reducing the lipid and moisture contents in rambutan seeds. Certainly, a high carbohydrate content in the substrate enhanced the POLS yield in the extract. Second, the defatting pretreatment ruptured the cells of rambutan seeds, eliminated the lipid barrier, and facilitated the release of POLS into the subcritical water. Therefore, the defatting pretreatment lowered the extraction temperature, reduced the liquid–solid ratio, and shortened the extraction time. However, the defatting pretreatment insignificantly affected the maximum values of total sugar content and monosaccharide profiles in the POLS-RS and POLS-DRS. Using the desirability function for the extraction of POLS from the DRS, the optimal conditions were determined to be a temperature in the range of 145–150 °C, an extraction time of 15 min, and a liquid–solid ratio of 10:1 (*w*/*w*). The maximum POLS yield and total sugar content were observed in the range of 52.33–55.63 g/100 g feedstock and 83.37–87.45 g/100 g POLS, respectively. The extracted POLS could be used as a thickener for making cream or as an extender in plant-based meat analogue products. The overall outcome of this work is the valorization of rambutan seeds would simultaneously reduce the amount of waste and generate additional profit for factories.

## Figures and Tables

**Table 1:** Box–Behnken design and observed values of POLS extraction yield and total sugar content. Numbers in parentheses are coded factor levels.

Run No.	Temperature (°C), X_1_	Time (min), X_2_	Liquid–Solid Ratio (*w*/*w*), X_3_	POLS Extraction Yield (g POLS/100 g Feedstock), (Y_1_)		Total Sugar (g Sugar/100 g POLS), (Y_2_)	
				RS (+1)	DRS (−1)	RS (+1)	DRS (−1)
1	120 (−1)	15 (−1)	20 (0)	14.172	57.079	42.464	42.673
2	180 (+1)	15 (−1)	20 (0)	23.075	31.862	49.810	92.621
3	120 (−1)	60 (+1)	20 (0)	23.833	53.641	59.271	69.285
4	180 (+1)	60 (+1)	20 (0)	7.281	41.887	41.117	74.731
5	120 (−1)	38 (0)	10 (−1)	21.340	49.990	68.174	69.696
6	180 (+1)	38 (0)	10 (−1)	13.621	13.255	35.886	93.591
7	120 (−1)	38 (0)	30 (+1)	21.036	35.480	73.183	55.422
8	180 (+1)	38 (0)	30 (+1)	30.091	21.937	78.982	95.293
9	150 (0)	15 (−1)	10 (−1)	40.528	54.955	49.715	82.964
10	150 (0)	60 (+1)	10 (−1)	28.033	28.641	86.475	99.511
11	150 (0)	15 (−1)	30 (+1)	32.659	43.219	77.603	87.209
12	150 (0)	60 (+1)	30 (+1)	39.606	46.246	90.284	84.374
13	150 (0)	38 (0)	20 (0)	37.374	48.984	92.610	71.515
14	150 (0)	38 (0)	20 (0)	33.051	47.156	89.285	64.409

**Table 2:** Proximate analysis of dried rambutan seed (RS) and defatted rambutan seed (DRS).

Source	Moisture (g/100 g)	Lipid (g/100 g)	Protein (g/100 g)	Carbohydrate (g/100 g)	Crude Fiber (g/100 g)	Ash (g/100 g)
RS	6.30 ± 0.05 ^a^	30.48 ± 0.49 ^a^	11.90 ± 0.14 ^a^	42.96 ± 0.15 ^b^	6.60 ± 0.25 ^a^	1.72 ± 0.02 ^a^
DRS	1.87 ± 0.02 ^b^	18.80 ± 0.20 ^b^	10.04 ± 0.22 ^a^	61.63 ± 0.74 ^a^	5.79 ± 0.11 ^a^	1.87 ± 0.01 ^a^

Different letters in the same column indicate statistically significant differences in means.

**Table 3:** Retention time and equivalent molecular weight (MWeq) of POLS-RS and POLS-DRS.

Sample	Retention Time (min)	MWeq (kDa)
POLS-RS	18.45	434.84 ± 3.07
	20.43	162.57 ± 0.00
	23.01	45.15 ±0.48
	23.76	30.29 ± 0.75
POLS-DRS	18.53	413.70 ± 8.77
	20.89	126.36 ± 4.02
	23.01	43.72 ± 1.54
	24.25	25.80 ± 2.37
	33.20	0.28 ± 0.00
	34.20	0.17 ± 0.00

**Table 4:** Monosaccharide profiles of POLS-RS and POLS-DRS.

Sample	Monosaccharide Content (mg/g POLS-RS)				
	Glu	Gal	Man	Rham	Ara
POLS-RS	919.07 ± 0.70 ^b^	64.63 ± 1.81 ^b^	N/D	3.60 ± 1.24 ^a^	12.70 ± 0.86 ^a^
POLS-DRS	922.34 ± 4.51 ^a^	66.87 ± 1.19 ^a^	N/D	2.65 ± 1.13 ^b^	8.14 ± 2.42 ^b^

Note: Glu is glucose, Gal is galactose, Man is mannose, Rham is rhamnose, Ara is arabinose, and N/D denotes not detected. Different letters in the same column indicate statistically significant differences in means.

## Data Availability

The original contributions presented in the study are included in the article/supplementary material, further inquiries can be directed to the corresponding author.

## Supplementary Materials

The following supporting information can be downloaded at: https://www.mdpi.com/article/10.3390/foods13131967/s1, Table S1: Fatty acid profile of rambutan seed lipids obtained by various extraction methods; Table S2: Analysis of variance the complete quadratic regression model in terms of coded units for POLS yield; Table S3: Analysis of variance the complete quadratic regression model in terms of coded units for total sugar (g/100 g POLS); Table S4: Numerical solutions of SWE for RS and DRS generated by Design Expert 13.0, sorting by high value of desirability when minimized temperature and extraction time are constraints; Table S5: Numerical solutions of SWE for RS and DRS generated by Design Expert 13.0, sorting by high value of desirability when all operating parameter had no constraint.
