# Supplementary material for: Effects of Defatting Pretreatment on Polysaccharide Extraction from Rambutan Seeds Using Subcritical Water: Optimization Using the Desirability Approach
Source: Foods. 2024 Jun 21;13(13):1967. doi: 10.3390/foods13131967 (PMC11241141; doi:10.3390/foods13131967)
Supplement: Supplementary file 1 [file foods-13-01967-s001.zip › foods-3054496-supplementary.pdf]

Table S1. Fatty acid profile of rambutan seed lipids obtained by various extraction methods.

Sample	Fatty acid content (g/100 g lipid)				
	C16:0	C18:0	C18:1	C18:2	C20:0
DS (Soxhlet)	4.02	N/D	47.04	0.37	40.34
SPC	2.87	N/D	49.56	0.06	44.41
SPC-$SCCO_2$	4.51	N/D	40.16	3.65	37.57

N/D, not detected.

Table S2. Analysis of variance the complete quadratic regression model in terms of coded units for POLS yield.

Source	Sum of Square	df	Mean Square	F-value	p-value
Model	4154.18	13	319.55	4.6	0.0039
X_1-Temperature	543.97	1	543.97	7.83	0.0142
X_2-Time	50.34	1	50.34	0.7246	0.409
X_3-L/S	22.13	1	22.13	0.3185	0.5814
X_4-Feedstock	1555.8	1	1555.8	22.39	0.0003
X_1X_2	18.12	1	18.12	0.2608	0.6175
X_1X_3	199.73	1	199.73	2.87	0.1121
X_1X_4	409.49	1	409.49	5.89	0.0293
X_2X_3	297.5	1	297.5	4.28	0.0575
X_2X_4	2.01	1	2.01	0.0289	0.8675
X_3X_4	24.6	1	24.6	0.3541	0.5613
X_1^2	878.23	1	878.23	12.64	0.0032
X_2^2	17.16	1	17.16	0.247	0.6269
X_3^2	106.65	1	106.65	1.53	0.2357
Residual	972.71	14	69.48		
Lack of Fit	961.7	12	80.14	14.55	0.066
Pure Error	11.01	2	5.51		
Cor Total	5126.89	27			
Model	4154.18	13	319.55	4.6	0.0039

*p-values more than 0.05 indicate a non-significant parameter.

Table S3. Analysis of variance the complete quadratic regression model in terms of coded units for total sugar (g/100 g POLS).

Source	Sum of Squares	df	Mean Square	F-value	p-value
Model	9122.68	16	570.17	14.57	< 0.0001
X_1-Temperature	434.82	1	434.82	11.11	0.0067
X_2-Time	399.9	1	399.9	10.22	0.0085
X_3-L/S	205.15	1	205.15	5.24	0.0428
X_4-Feedstock	524	1	524	13.39	0.0038
X_1X_2	613.78	1	613.78	15.69	0.0022
X_1X_3	365.34	1	365.34	9.34	0.0109
X_1X_4	1529.92	1	1529.92	39.1	< 0.0001
X_2X_3	234.27	1	234.27	5.99	0.0324
X_2X_4	77.09	1	77.09	1.97	0.188
X_3X_4	666.51	1	666.51	17.03	0.0017
X_1^2	1582.25	1	1582.25	40.44	< 0.0001
X_2^2	136.81	1	136.81	3.5	0.0883
X_3^2	364.53	1	364.53	9.32	0.011
$X_1^2 X_4$	868.5	1	868.5	22.2	0.0006
$X_2^2X_4$	722.46	1	722.46	18.46	0.0013
$X_3^2X_4$	319.5	1	319.5	8.17	0.0156
Residual	430.44	11	39.13		
Lack of Fit	399.66	9	44.41	2.89	0.2838

*p-values more than 0.0500 indicate a non-significant parameter.

Table S4. Numerical solutions of SWE for RS and DRS generated by Design Expert 13.0, sorting by high value of desirability when minimized temperature and extraction time are constraints.

No.	Temperature (°C)	Extraction time (min)	L/S	Feedstock	PLOS yield (g/100 g feedstock)	Total sugar (g/100 g PLOS)	Desirability
1	128	15	10	DRS	58.68	74.32	0.85
2	128	15	10	DRS	58.69	74.18	0.85
3	129	15	10	DRS	58.67	74.42	0.85
4	129	15	10	DRS	58.64	74.75	0.85
5	128	15	10	DRS	58.72	73.80	0.85
6	127	15	10	DRS	58.73	73.55	0.85
7	130	15	10	DRS	58.59	75.19	0.85
8	127	15	10	DRS	58.74	73.35	0.85
9	130	15	10	DRS	58.56	75.38	0.85
10	126	15	10	DRS	58.76	72.66	0.85
11	128	15	10	DRS	58.73	73.91	0.85
12	126	15	10	DRS	58.76	72.24	0.85
13	124	15	10	DRS	58.73	71.16	0.85
14	128	15	10	DRS	58.86	73.73	0.85
15	123	15	10	DRS	58.64	70.05	0.85
16	135	15	10	DRS	57.79	78.78	0.85
17	128	16	10	DRS	58.35	74.13	0.85
18	134	15	10	DRS	57.90	78.05	0.85
19	135	15	10	DRS	57.69	79.07	0.85
20	130	16	10	DRS	58.13	75.17	0.85
21	121	15	10	DRS	58.44	68.51	0.85
22	131	16	10	DRS	58.22	75.86	0.85
23	120	15	10	DRS	58.35	67.94	0.85
24	127	16	10	DRS	58.12	73.02	0.85
25	120	15	10	DRS	58.31	67.76	0.85
26	130	16	10	DRS	57.91	75.34	0.85
27	138	15	10	DRS	56.97	80.90	0.84
28	128	17	10	DRS	57.93	73.96	0.84
29	129	17	10	DRS	57.86	74.24	0.84
30	120	16	10	DRS	57.84	67.57	0.84
31	133	15	11	DRS	58.95	75.59	0.84
32	134	15	11	DRS	58.58	76.90	0.84
33	121	15	10	DRS	58.78	67.21	0.84
34	133	17	10	DRS	57.00	77.60	0.84

No.	Temperature (°C)	Extraction time (min)	L/S	Feedstock	PLOS yield (g/100 g feedstock)	Total sugar (g/100 g PLOS)	Desirability
35	131	18	10	DRS	57.18	75.80	0.84
36	140	15	10	DRS	56.05	82.68	0.83
37	133	19	10	DRS	56.42	76.86	0.83
38	130	20	10	DRS	56.26	74.76	0.82
39	120	15	11	DRS	59.65	63.16	0.81
40	144	15	10	DRS	54.24	85.31	0.81
41	126	15	12	DRS	60.96	65.78	0.81
42	141	15	12	DRS	58.56	76.98	0.81
43	120	15	12	DRS	60.12	61.42	0.80
44	151	15	10	DRS	50.69	88.96	0.77
45	147	15	18	DRS	58.94	69.84	0.74
46	144	15	20	DRS	59.30	65.79	0.74
47	144	15	22	DRS	57.00	65.74	0.73
48	144	15	22	DRS	57.00	65.94	0.73
49	143	15	22	DRS	57.00	65.26	0.73
50	145	15	22	DRS	57.00	66.40	0.73
51	143	15	23	DRS	57.00	64.87	0.73
52	146	15	22	DRS	57.00	68.01	0.73
53	140	15	23	DRS	57.00	62.56	0.73
54	142	15	25	DRS	53.09	66.13	0.73
55	142	15	25	DRS	53.06	65.37	0.73
56	141	15	29	DRS	43.79	71.23	0.72
57	144	27	30	RS	32.67	91.94	0.66
58	144	26	30	RS	32.58	91.55	0.66
59	144	27	30	RS	32.72	92.27	0.66
60	144	26	30	RS	32.51	91.50	0.66
61	144	26	30	RS	32.71	91.74	0.66
62	144	26	30	RS	32.41	91.26	0.66
63	145	27	30	RS	33.00	92.83	0.66
64	143	26	30	RS	32.30	91.23	0.66
65	145	27	30	RS	33.03	92.48	0.66
66	145	26	30	RS	33.06	92.10	0.66
67	144	28	30	RS	33.15	93.46	0.66
68	144	25	30	RS	32.05	90.02	0.66
69	144	23	30	RS	31.66	87.22	0.66
70	140	27	30	RS	30.92	89.40	0.66

No.	Temperature (°C)	Extraction time (min)	L/S	Feedstock	PLOS yield (g/100 g feedstock)	Total sugar (g/100 g PLOS)	Desirability
71	143	32	30	RS	33.55	95.84	0.66
72	138	24	10	RS	35.43	69.87	0.64
73	138	24	10	RS	35.38	70.05	0.64
74	138	24	10	RS	35.44	69.52	0.64
75	138	25	10	RS	35.22	70.43	0.64
76	138	23	10	RS	35.51	68.92	0.64
77	140	23	10	RS	36.03	68.62	0.64
78	139	22	10	RS	36.10	67.64	0.63

Table S5. Numerical solutions of SWE for RS and DRS generated by Design Expert 13.0, sorting by high value of desirability when all operating parameter had no constraint.

No.	Temperature (°C)	Extraction time (min)	L/S	Feedstock	PLOS yield (g/100 g feedstock)	Total sugar (g/100 g PLOS)	Desirability
1	145	15	10	DRS	53.71	85.96	0.86
2	146	15	10	DRS	53.55	86.14	0.86
3	146	15	10	DRS	53.47	86.23	0.86
4	144	15	10	DRS	54.20	85.36	0.86
5	146	15	10	DRS	53.45	86.17	0.86
6	143	15	10	DRS	54.75	84.65	0.86
7	148	15	10	DRS	52.47	87.30	0.86
8	145	15	10	DRS	53.63	85.91	0.86
9	148	15	10	DRS	52.33	87.45	0.86
10	145	15	10	DRS	53.86	85.55	0.86
11	145	15	10	DRS	54.37	84.87	0.86
12	145	15	10	DRS	53.54	85.80	0.86
13	141	15	10	DRS	55.63	83.37	0.86
14	145	16	10	DRS	53.56	85.49	0.86
15	142	16	10	DRS	54.88	83.89	0.85
16	140	15	10	DRS	56.11	82.58	0.85
17	147	15	11	DRS	53.91	84.95	0.85
18	146	16	10	DRS	52.87	86.01	0.85
19	146	15	11	DRS	54.64	83.78	0.85
20	139	15	10	DRS	56.58	81.71	0.85
21	156	60	30	DRS	45.70	90.75	0.81
22	156	60	30	DRS	45.75	90.68	0.81
23	156	60	30	DRS	45.64	90.85	0.81
24	156	60	30	DRS	45.83	90.55	0.81
25	155	60	30	DRS	45.85	90.52	0.81
26	156	60	30	DRS	45.76	90.66	0.81
27	157	60	30	DRS	45.51	91.04	0.81
28	157	60	30	DRS	45.44	91.14	0.81
29	156	60	30	DRS	46.23	89.90	0.81
30	154	60	30	DRS	46.02	90.21	0.81
31	156	60	30	DRS	46.20	89.91	0.81
32	156	60	30	DRS	45.63	90.73	0.81
33	160	15	13	DRS	47.85	86.38	0.80
34	149	53	30	RS	40.03	99.00	0.80

No.	Temperature (°C)	Extraction time (min)	L/S	Feedstock	PLOS yield (g/100 g feedstock)	Total sugar (g/100 g PLOS)	Desirability
35	148	53	30	RS	40.03	99.00	0.80
36	148	53	30	RS	40.02	99.00	0.80
37	149	53	30	RS	40.11	98.79	0.80
38	149	53	30	RS	40.01	99.00	0.80
39	150	53	30	RS	40.00	99.00	0.80
40	149	53	30	RS	40.10	98.78	0.80
41	149	54	30	RS	40.16	98.64	0.80
42	148	54	30	RS	40.21	98.43	0.80
43	149	53	30	RS	39.90	99.36	0.80
44	152	52	30	RS	39.87	99.00	0.80
45	149	52	30	RS	39.84	99.52	0.80
46	151	60	26	DRS	51.59	80.92	0.80
47	152	52	30	RS	39.80	99.00	0.80
48	149	56	30	RS	40.70	96.87	0.80
49	149	57	30	RS	40.92	96.04	0.79
50	150	52	30	RS	39.43	99.00	0.79
51	164	60	28	DRS	45.68	87.64	0.79
52	151	52	30	RS	39.27	99.00	0.79
53	159	15	17	DRS	52.16	79.23	0.79
54	153	60	24	DRS	52.45	78.80	0.79
55	154	54	30	RS	39.96	96.99	0.79
56	144	60	30	DRS	46.69	85.52	0.79
57	157	49	30	RS	38.89	99.00	0.78
58	157	49	30	RS	38.83	99.23	0.78
59	155	48	30	RS	38.76	100.22	0.78
60	144	49	30	RS	38.53	100.03	0.78
61	156	45	30	RS	38.35	100.71	0.78
62	152	60	22	DRS	52.79	77.18	0.78
63	159	50	30	RS	38.54	97.35	0.77
64	147	60	20	DRS	53.15	76.18	0.77
65	160	44	30	RS	37.70	99.00	0.77
66	144	60	16	DRS	50.38	78.70	0.77
67	144	60	17	DRS	50.59	78.48	0.77
68	144	60	16	DRS	50.27	78.83	0.77
69	143	60	16	DRS	49.62	79.52	0.77
70	140	49	30	RS	37.49	99.00	0.77

No.	Temperature (°C)	Extraction time (min)	L/S	Feedstock	PLOS yield (g/100 g feedstock)	Total sugar (g/100 g PLOS)	Desirability
71	141	60	14	DRS	47.72	81.62	0.77
72	142	60	14	DRS	46.60	82.96	0.76
73	152	40	30	RS	37.40	101.77	0.76
74	157	37	30	RS	37.15	100.37	0.76
75	159	15	24	DRS	48.00	79.66	0.75
76	144	35	10	RS	33.37	81.25	0.60
77	144	35	10	RS	33.43	81.11	0.60
78	144	35	10	RS	33.41	81.15	0.60
79	144	34	10	RS	33.51	80.96	0.60
80	145	35	10	RS	33.43	81.11	0.60
81	145	34	10	RS	33.59	80.77	0.60
82	145	35	10	RS	33.32	81.30	0.60
